# Gastrocolic fistula secondary to adenocarcinoma of the transverse colon: a case report

**DOI:** 10.1186/s13256-015-0725-2

**Published:** 2015-10-27

**Authors:** Omar Vergara-Fernández, Ylse Gutiérrez-Grobe, María Lavenant-Borja, Carlos Rojas, Nahum Méndez-Sánchez

**Affiliations:** 1 Colorectal Surgery, Medica Sur Clinic and Foundation, Puente de Piedra 150, Col. Toriello Guerra, Mexico City, 14050 Mexico; 2 Liver Research Unit, Medica Sur Clinic and Foundation, Puente de Piedra 150, Col. Toriello Guerra, Mexico City, 14050 Mexico

**Keywords:** Adenocarcinoma, Colon, En bloc, Resection, Gastrectomy, Gastrocolic fistula

## Abstract

**Introduction:**

Gastrocolic fistula is a rare complication of adenocarcinoma of the colon. Despite radical resections, these patients usually have a poor prognosis with a mean survival of 23 months and long-term survival is rarely reported.

**Case presentation:**

A 48-year-old Latino-American man presented with watery diarrhea, diffuse abdominal pain and weight loss for 3 months. A computed tomography scan revealed a mass in the splenic flexure that had infiltrated his stomach and diaphragm. Panendoscopy and colonoscopy confirmed the presence of a fistula between the distal transverse colon and the stomach, which was secondary to a colon cancer. His colon, stomach and left diaphragm were resected en bloc. A histological examination revealed a moderately differentiated adenocarcinoma of the colon that had infiltrated the full width of the gastric wall with 37 negative lymph nodes and clear surgical margins. Adjuvant chemotherapy with capecitabine and oxaliplatin was administered after surgery. Our patient is alive and without any recurrence 5 years after surgery.

**Conclusions:**

En bloc resection with adjuvant chemotherapy offers the best treatment option for gastrocolic fistulas. This is one of the patients with greater survival reported in the medical literature.

## Introduction

Cologastric or gastrocolic fistula is a pathologic communication between a segment of the colon and the stomach, and may be caused by benign or malignant diseases of the gastrointestinal tract. It has been associated with diseases such as gastric tumors [1–3], gastric ulcers [4, 5] and pancreatitis [6]. Symptoms tend to be nonspecific, but most patients present with a triad of diarrhea, weight loss and feculent vomiting. Gastrocolic fistula secondary to colon cancer is very rare. We present the case of a patient with a gastrocolic fistula originating from transverse colon cancer and treated with a radical en bloc surgery and adjuvant chemotherapy.

## Case presentation

A 48-year-old Latino-American man was admitted to our hospital because of a 3-month history of intermittent watery diarrhea. Initially, the diarrhea was self-limited, and the stools did not contain mucus or blood. One week before his admission, the diarrhea became persistent and was accompanied by black, tarry, malodorous feces. In addition, our patient had a dull abdominal pain, predominantly in the left upper quadrant, which radiated to the lumbar region (the renal fossa). Our patient had no family history of colon cancer. He smoked one pack of cigarettes daily and occasionally consumed alcohol. His weight had decreased by 10 kg during the last 6 weeks before his admission. He had no history of peptic ulcer disease, inflammatory bowel disease, trauma or abdominal surgery. His body temperature was 37 °C, heart rate was 70 beats per minute, respiration rate was 19 breaths per minute, and blood pressure was 100/70 mmHg.

Our patient experienced abdominal pain in response to deep palpation of the left upper quadrant of his abdomen. There were no palpable masses. Auscultation revealed an elevated frequency of bowel sounds. His hemoglobin level was 9.2 g/dL, hematocrit was 28.2 %, white blood cell count was 8,900/mm^3^, and platelet count was 623,000/mm^3^. His lactate dehydrogenase concentration was 121 U/L, and his gamma glutamyl transpeptidase level was elevated (71 U/L). His albumin level was low (2.4 g/dL). Other blood chemical and enzyme values were normal. An electrocardiogram showed no abnormalities.

**Fig. 1:**
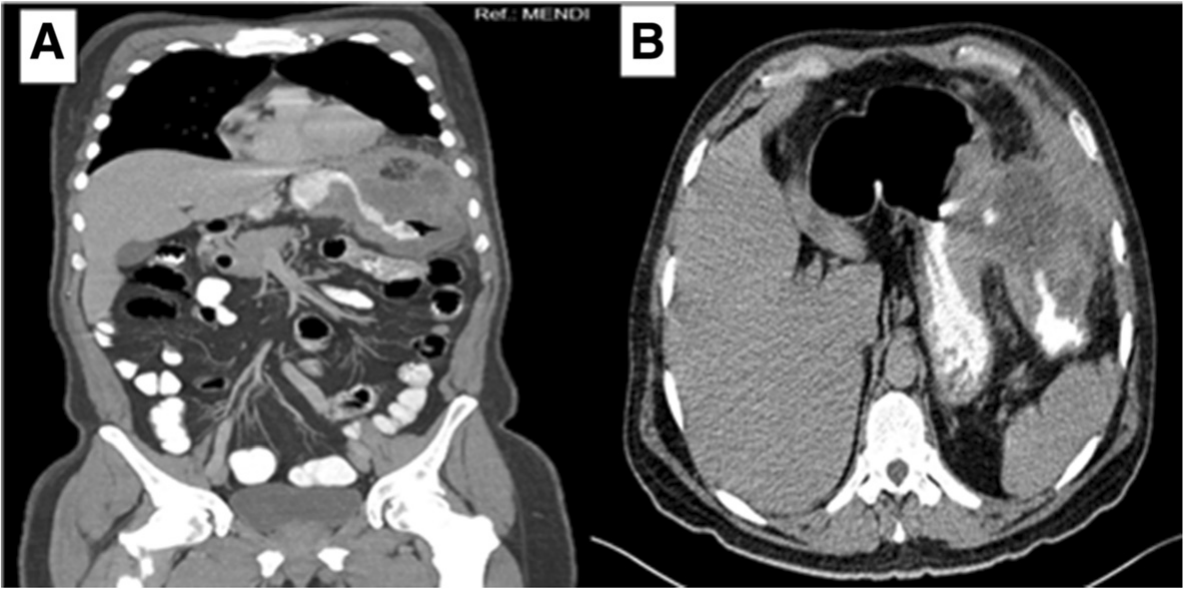
**a** Coronal computed tomography image showing a gastrocolic fistula secondary to a tumor of the splenic flexure of the colon infiltrating the greater curvature of the stomach and diaphragm. **b** Computed tomography image of the abdomen showing passage of contrast material through a fistulous connection between the stomach and the splenic flexure of the colon

**Fig. 2:**
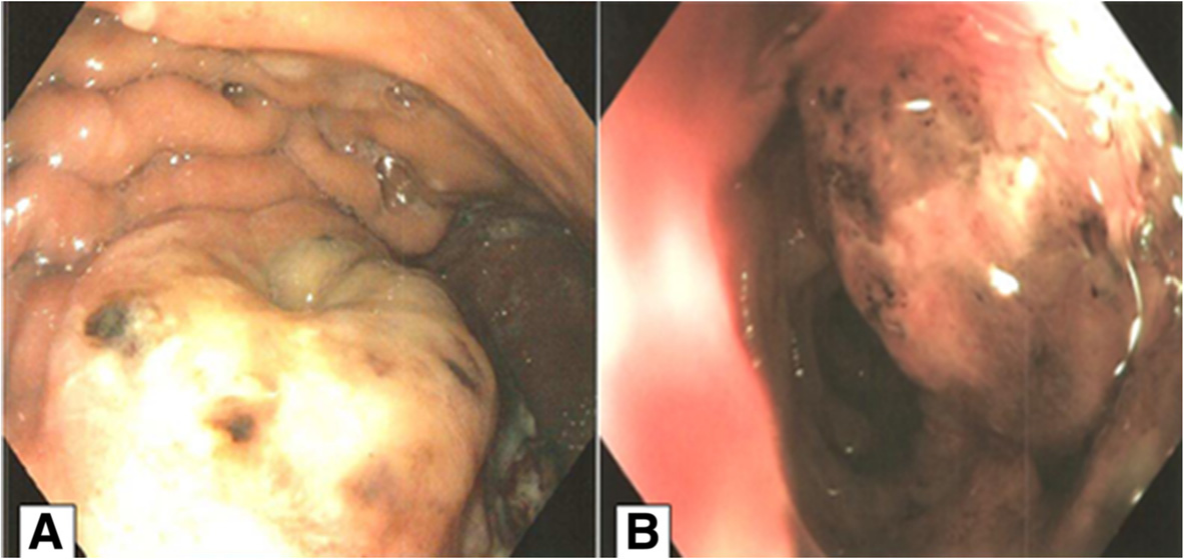
**a** Gastroscopy image showing an ulcerated mass at the body of the stomach and the opening of a fistula above the mass. **b** A mass at the splenic flexure of the colon

**Fig. 3:**
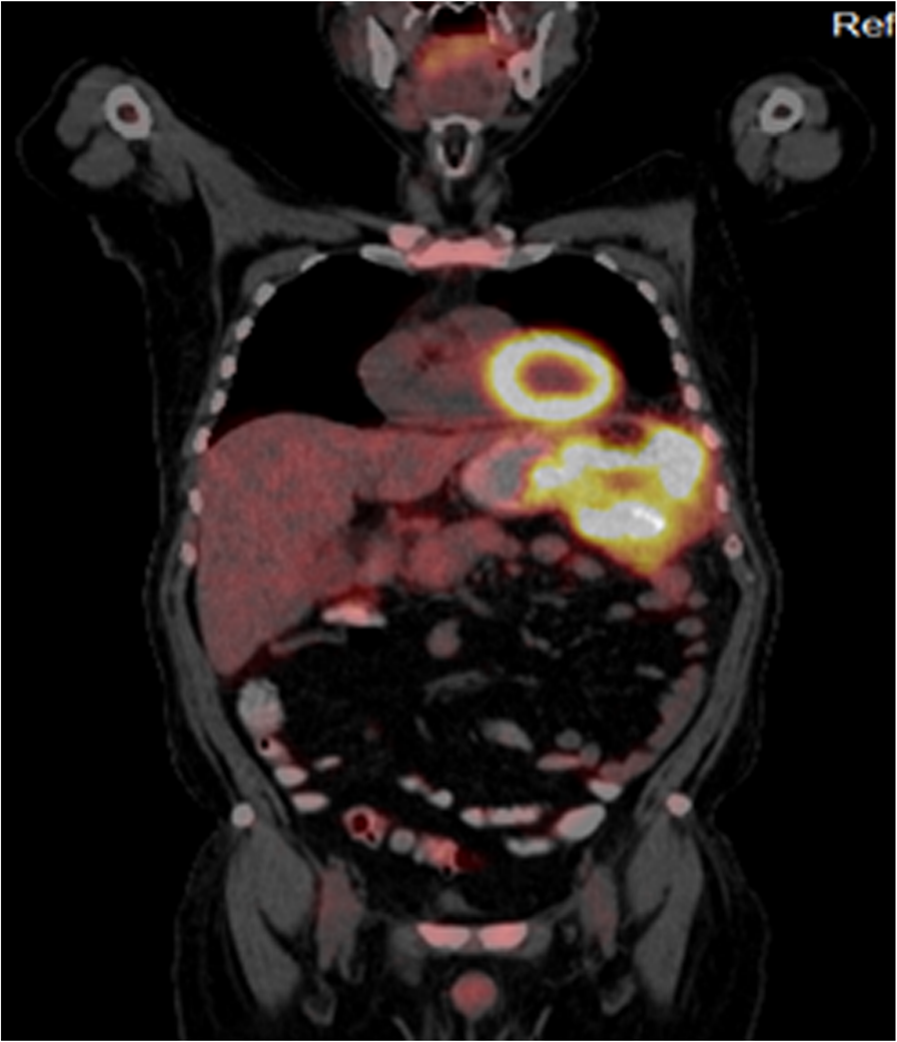
Thoracoabdominal positron emission tomography image showing a splenic flexure tumor with enhancement of the greater curvature of the stomach and diaphragm

A computed tomography (CT) scan of his abdomen showed a mass in the splenic flexure of the colon that had infiltrated the greater curvature of the stomach and revealed the presence of a gastrocolic fistula; the mass also involved the left anterolateral region of the diaphragm (Fig. 1). The endoscopy revealed an ulcerated polypoid gastric neoplasm in the greater curvature of the stomach and an ulcerated colonic neoplasm in the splenic flexure of the colon and the distal part of the transverse colon. The largest diameter of the ulcer was 1.4 cm (Fig. 2). Biopsies taken from his stomach and colon revealed a moderately differentiated adenocarcinoma of intestinal type originating from a villous adenoma and a moderately differentiated adenocarcinoma originating from a villous adenoma, respectively. A positron emission tomography (PET) scan performed for staging purposes before surgery suggested involvement of the gastric, hepatic and preaortic lymph nodes (Fig. 3).

**Fig. 4:**
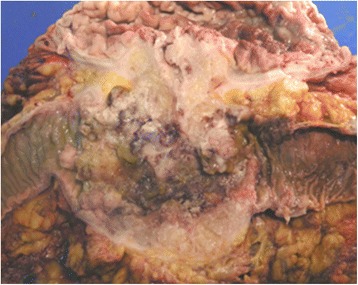
Surgical specimen showing the fistula between the stomach and the transverse colon

**Fig. 5:**
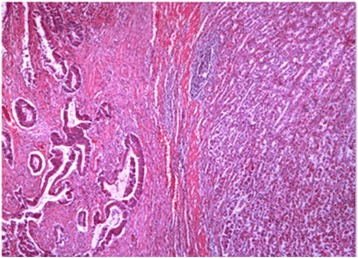
A colon adenocarcinoma infiltrating the gastric wall

In view of these findings, a radical en bloc resection was performed involving a subtotal gastrectomy, a transverse left-side colectomy, small bowel resection, and left diaphragm (Fig. 4). Histology revealed a moderately differentiated adenocarcinoma that had originated from a villous adenoma and infiltrated the full width of the gastric wall (Fig. 5). Thirty-seven lymph nodes and surgical margins were free of tumor. The Dukes´ classification of the tumor was B. Our patient developed a subphrenic abscess that was treated with a CT-guided drainage. Our patient received adjuvant chemotherapy consisting of six courses of capecitabine (2 g/day for 14 days) and oxaliplatin (100 mg/day for 15 days). Our patient has now survived for 5 years. Colonoscopies were performed at 1, 3 and 5 years. Chest/abdominal/pelvic CT scans were carried on annually for 5 years. Fluorodeoxyglucose (FDG)-PET scans were performed at 3 and 5 years of follow-up. These studies have shown no evidence of recurrence.

## Discussion

Gastrocolic fistula is an uncommon complication of benign and malignant diseases of the gastrointestinal tract [7]. In the past, neoplasms of the gastrointestinal tract, such as adenocarcinoma of the transverse colon (Western countries) and adenocarcinoma of the stomach (Japan), were the most common etiologies of gastrocolic fistula [8]. At present, gastrocolic fistulas secondary to colon carcinoma are rare, possibly due to earlier diagnosis of colon cancer. Two theories have been proposed for its development: the cancerous gastrocolic fistulas appear to arise from direct extension of the tumor across the gastrocolic omentum; another theory proposes that a tumor ulcer might cause an inflammatory peritoneal reaction leading to adherence and fistula formation [7, 9].

The classic clinical presentation of a gastrocolic fistula is a triad of diarrhea, weight loss and feculent vomiting [8]. Other symptoms are abdominal pain, feculent eructation, fatigue and nutritional deficiencies [10]. In our case, abdominal pain, diarrhea and weight loss were the main symptoms. Our patient had also upper gastrointestinal bleeding because of ulceration of the greater curvature of the stomach. Some authors have suggested that a barium enema is the most sensitive method for detecting this type of fistula due to the direction of the flow from the fistula is mostly from the colon to the stomach. In our case, the first radiological assessment was CT, which delineated the fistula extending from the colon to the stomach. Upper endoscopy is an excellent tool for visualizing the fistula opening and for taking biopsy samples for histopathology examination.

Surgical options for gastrocolic fistula have changed over time, from second- and third-stage surgeries in the 1940s to the current one-stage en bloc resection technique [11]. Practice parameters of the American Society of Colon and Rectal Surgeons stated that at the time of surgery, it is impossible to distinguish between malignant and inflammatory adhesions, and recommend that colon cancer adherent to adjacent organs should be resected en bloc [12]. In a Canadian study, the principles of en bloc resection was violated in more than 50 % of eligible locally advanced adherent colorectal cancers in two provinces, and physicians with fewer years in practice were more likely to perform multivisceral resections than older physicians [13]. Hunter *et al*., reported that 5-year survival after en bloc resection of colon cancer is higher compared to colectomy with separation of adherent organs (61 % versus 23 %) [14]. In a German study, local recurrence and overall 5-year survival for multivisceral resection for locally advanced colorectal cancer were 11 % and 51 %, respectively [15].

In a Japanese report of 14 gastrocolic fistulas, mean age was 50 years with a male to female ratio of 10:4; it is similar to our patient, who is male, and 48 years old. The fistulous formation between the distal half of the transverse colon and the greater curvature of the stomach was seen in 13 of 14 cases (93 %), similar to ours. These patients usually have a poor prognosis; the mean survival is 23.4 months (range, 3 to 112) [8]. As far as we know, the longest disease-free survival reported in the medical literature for a malignant gastrocolic fistula belongs to a 24-year-old woman, who survived for more than 10 years [10]. Our patient has now survived for 5 years, and has no evidence of tumor recurrence.

## Conclusions

Gastrocolic fistula is a rare complication of adenocarcinoma of the colon. En bloc resection with adjuvant chemotherapy offers the best treatment option for gastrocolic fistulas. This is one of the patients with longest survival reported in the medical literature.

## Consent

Written informed consent was obtained from the patient for publication of this case report and any accompanying images. A copy of the written consent is available for review by the Editor-in-Chief of this journal.

## Abbreviations

CT: computed tomography scan
FDG: fluorodeoxyglucose
PET: positron emission tomography
